# Ursodeoxycholic Acid for the Prevention of Relapse of Pregnancy-Related Acute Gallstone Pancreatitis

**DOI:** 10.3390/jcm15072580

**Published:** 2026-03-27

**Authors:** Alberto Maringhini, Rosalia Patti, Marco Maringhini, Jacopo Maringhini

**Affiliations:** 1 Pancreatic Unit, La Maddalena Hospital, Via San Lorenzo 312D, 90146 Palermo, Italy; 2 Pancreatic Unit, ARNAS Civico Hospital, 90127 Palermo, Italy; rosalia.patti@arnascivico.it; 3 Internal Medicine, ARNAS Civico, 90127 Palermo, Italy; marcomaringhini@gmail.com; 4 School of Medicine, University of Palermo, 90127 Palermo, Italy; jacopugno@gmail.com

**Keywords:** gallstones, biliary sludge, acute pancreatitis, pregnancy, cholecystectomy, ursodeoxycholic acid, breastfeeding

## Abstract

**Introduction:** Acute gallstone pancreatitis is a potentially severe disease associated with morbidity and mortality. Cholecystectomy is recommended to prevent recurrence. During pregnancy, surgical management is challenging, and in the post-partum period small gallstones may spontaneously disappear. Ursodeoxycholic acid (UDCA) is safe during the last 6 months of pregnancy and effective in dissolving small gallstones, although recurrence after discontinuation is common in the general population. The optimal strategy to prevent recurrent acute pancreatitis during and after pregnancy remains unclear. **Methods:** Between 2002 and 2017 we prospectively treated women with acute pancreatitis related to small gallstones (≤1 cm in diameter) during the last six months of pregnancy or within the first post-partum year who declined surgery. Patients received UDCA until stone dissolution. A patent cystic duct was confirmed by ultrasonography; after delivery, a non-contrast CT scan was performed to exclude calcified stones. Patients were followed for at least 6 years or until recurrence, with serial clinical and ultrasonographic examinations. **Results:** UDCA was associated with complete dissolution in 13/14 women within a mean ± SD of 7.77 + 3.1 months. One patient experienced gallstone recurrence 75 months after treatment discontinuation. Two patients developed recurrent pancreatitis (at 1 and 88 months respectively). Twelve women remained free of recurrence over a mean ± SD follow-up of 79.5 + 9.4 months. **Discussion**: This is an observational study in which we document that UDCA may facilitate the spontaneous dissolution of small gallstones after delivery and can be considered a bridge strategy during pregnancy when surgery is not feasible. However, this study cannot determine the additional benefit of UDCA over the spontaneous disappearance of stones observed after delivery because we had no control group. Cholecystectomy remains the standard of care post-partum. Medical therapy should be reserved for women who refuse surgery and it requires close ultrasonographic surveillance. The main strength of this study is the prospective long-term follow-up of a consecutive cohort with a rare condition. Limitations include the small sample size, missing control group and single-center design.

## 1. Introduction

In a recent population-based cohort study of Sicilian women of childbearing age, the incidence of acute pancreatitis during pregnancy was similar to that in non-pregnant women. This incidence increases markedly during the first 6 months after delivery before gradually returning to the baseline over two years [1]. This increase is primarily associated with gallstones, and it is more pronounced in younger women [1,2].

Biliary sludge and stones are a well-established cause of acute pancreatitis [3,4]. Women with gallstones have a 12 to 25 times greater risk of developing acute pancreatitis compared with the general population [5]. Cholecystectomy reduces the risk of recurrence by 6- to 12-fold [5].

During pregnancy, new sludge and stones develop in approximately 31% and 2% of women, respectively [6]. In the first post-partum year spontaneous disappearance occurs in 68.3% of sludge and 30% of gallstone cases, especially in older mothers, which explains why the increased incidence of acute pancreatitis affects mainly younger women [6,7].

Cholecystectomy can be performed during pregnancy, preferably in the second trimester and in tertiary hospitals with specific expertise [8]. However, in many settings it remains challenging.

Ursodeoxycholic acid (UDCA) is safe in the last six months of pregnancy [9] and effective in dissolving sludge and small cholesterol gallstones [10]. Since pregnancy-related acute pancreatitis is rare, randomized controlled trials are unlikely. We therefore conducted a prospective observational study of women with small gallstone-related acute pancreatitis during pregnancy or within the first post-partum year who refused surgery and were treated with UDCA.

## 2. Methods

Between 2002 and 2017 we enrolled all consecutive women with acute pancreatitis and gallstones (≤10 mm) occurring in the last six months of pregnancy or within the first post-partum year.

Acute pancreatitis was diagnosed according to the revised Atlanta criteria [11] when at least two of the following were present:

1. Acute onset of persistent and severe epigastric pain radiating to the back.
2. Serum lipase or amylase activity > 3 × the upper limit of normal.
3. Characteristic imaging findings on contrast-enhanced CT, MR or transabdominal ultrasonography (US).

Gallstones were diagnosed by US as a mobile echogenic structure (>2 mm) with positional change casting acoustic shadows.

Patients with gallstones > 1.0 cm were excluded. Surgery was proposed to all women: only those who refused were enrolled. Written informed consent was obtained, and the study was approved by the Institutional Ethics Committee.

UDCA (10 mg/kg/day) was started after recovery from acute pancreatitis in patients with a patent cystic duct on US. After delivery, a non-contrast CT scan was performed to exclude stone calcification. Follow-up included clinical examination and gallbladder US (every 3 months during year 1, every 6 months during year 2, then annually). UDCA was discontinued 3 months after complete stone disappearance. Patients were followed up for at least 6 years.

## 3. Results

Fourteen women were recruited: 5 during the last six months of pregnancy and 9 post-partum (Table 1). The mean age + SD was 25.9 + 6.5 years. Seven additional women were excluded: five underwent cholecystectomy after delivery and two had gallstones > 1.0 cm.

All pregnancy deliveries were uncomplicated; no child malformations were observed. All cases of acute pancreatitis were mild except one (patient n. 5), in which a patient developed pancreatic effusion at 7 months of pregnancy. She was lost to follow-up after 66 months with an empty gallbladder but was later readmitted 18 months afterward with recurrent acute pancreatitis and gallstones (*n* = 3, largest 6 mm); she then underwent cholecystectomy without complications. Another patient developed recurrent acute pancreatitis one month after discharge, before dissolution could occur, and she was operated on uneventfully.

In the remaining 13 women, gallstone dissolution was achieved after a mean + SD of 7.8 + 3.1 months.

In 12 women, UDCA was associated with complete stone dissolution with no recurrence over a mean ± SD follow-up of 79.5 ± 9.4 months.

## 4. Discussion

This study demonstrates that UDCA is associated with dissolution of small gallstones and with the prevention of recurrent acute pancreatitis in most cases (12/14 women over 79.5 + 9.35 months of follow-up). However, this study cannot determine the additional benefit of UDCA over the spontaneous disappearance of stones observed after delivery because we had no control group, although our gallstone dissolution rate is higher than the 30% observed mainly in women older than 35 years old [6]. Failure occurred in one patient with early recurrence, likely due to insufficient time for dissolution, and one with late recurrence after apparent stone clearance.

We report a consecutive series of patients with acute pancreatitis during pregnancy or in the first year post-partum. Seven women were excluded: five because they underwent cholecystectomy post-partum after acute pancreatitis, and two because they had gallstones > 1.0 cm.

All deliveries and newborn outcomes were complication-free; we did not observe any fetal malformation and there was no maternal mortality due to acute pancreatitis. Only one woman had local complication of acute pancreatitis (peripancreatic fluid collection). To our knowledge this is the first prospective observational study evaluating medical gallstone dissolution to prevent recurrent pancreatitis during pregnancy or in the first year after delivery. UDCA appeared to perform better than in the general population, likely because gallstones associated with pregnancy-related pancreatitis are not only small but also recently formed. Moreover, pregnancy-related changes in bile composition and gallbladder motility may favor stone clearance; UDCA may therefore facilitate the spontaneous dissolution of sludge and small stones in the post-partum period.

The main limitations of the study are the small sample size, which was inevitable given the rarity of the condition, and the single-center design. We had no control group, so we could not define the exact role of UDCA in dissolving gallstones because of the known spontaneous disappearance of small stones in the post-partum period. In addition, five potentially eligible patients were not included because they underwent cholecystectomy in accordance with international guidelines. Therefore, our findings apply only to women in the last six months of pregnancy or in the first post-partum year, with small stones (≤10 mm), a patent cystic duct and no calcified gallstones on non-contrast CT scan. The mean age of participants was 25.9 + 6.5. This is consistent with epidemiological evidence showing that post-partum biliary pancreatitis occurs more frequently in younger women [1,2].

Recent European and American guidelines, including those from American College of Gastroenterology and the guidelines supported by American Pancreatic Association, European Pancreatic Club, Indian Pancreas Club and Japan Pancreas Society, recommend early cholecystectomy after mild acute pancreatitis to prevent recurrence (strong evidence) [12,13,14]. Only the latter addressed acute pancreatitis during pregnancy and recommends early cholecystectomy for pregnant patients with mild acute biliary pancreatitis, preferably in the second and early third trimester (strong recommendation; moderate-quality evidence) [14].

Although laparoscopic cholecystectomy is considered safe in the second and early third trimester, it should ideally be performed in a tertiary hospital with specific expertise. In real-world practice, access to appropriately experienced surgical teams may be limited. In such circumstances, UDCA treatment may represent a pragmatic bridging strategy to defer cholecystectomy until after delivery.

The use of UDCA for gallstone dissolution has declined due to concerns about long-term recurrence (30–50% within 5 years) [10]. However, in the post-partum period small gallstones may spontaneously dissolve. In our cohort UDCA was associated with gallstone dissolution in 13/14 women (92.9%) within a mean of 7.8 months. The only patient without dissolution discontinued therapy after one month due to recurrent pancreatitis. Among those with documented dissolution, only one of 13 (7.7%) developed stone recurrence after 74 months without medical therapy. Overall, only 2 of 14 (12.9%) experienced recurrent pancreatitis.

The association between gallstones and acute pancreatitis has been recognized since the early 20th century, when Opie described a patient who had acute pancreatitis associated with gallstones impacted in the ampulla of Vater [15]. Subsequent studies demonstrated that a large proportion of patients with suspected biliary pancreatitis pass stones in stool in the first days after symptom onset [4,16,17]. Other reports have documented gallstones in the common bile duct in a minority of patients and gallbladder stones in the majority [18]. Microscopic biliary sludge has also been detected in duodenum juice in a substantial fraction of cases labeled as idiopathic pancreatitis [3].

Epidemiological data from Olmsted County indicate that women with gallstones have a 25 and 12 times higher maximal and minimal relative risk of acute pancreatitis, respectively, compared with the general population. In this study cholecystectomy reduced the risk by 6- to 12-fold [5].

In a population-based cohort study the acute pancreatitis incidence during pregnancy was 21.6/100,000 persons-year, not different from the 20.0/100,000 persons-years in non-pregnant women of childbearing age. In the post-partum period acute pancreatitis increased to 96.4 in the first semester, decreasing to 26.1/100,000 only in the third year after delivery. Acute pancreatitis was related to gallstones in 38% of cases during pregnancy, in 70% of cases in post-partum women and in 49% of cases in non-pregnant women. In this cohort, acute pancreatitis incidence increased with age in non-pregnant women, whereas it decreased with age in post-partum women [1].

During pregnancy the incidence of new sludge and stones is 31% and 2%, respectively [6], but in the first year after delivery, they spontaneously disappear in 68.3% and 30% of cases, respectively [6,7]. During pregnancy obesity is a risk factor for new sludge; the presence of sludge is also a risk factor for new gallstones [6]. After delivery, an age < 30 years and a small diameter (<8 mm) of gallstones are predictors of spontaneous disappearance [6,7].

This phenomenon—new stones during pregnancy, spontaneous disappearance in the first year after delivery of small stones—especially in older mothers (>30 years), confirms old epidemiological studies. These studies demonstrated that parity is a risk factor only for women who become pregnant at a younger age [19,20]. This evidence also suggests that most disappeared stones do not relapse without any treatment.

The precipitation of biliary sludge and stones during pregnancy is due to increased bile cholesterol saturation for high serum estrogen levels and reduced gallbladder motility caused by high serum progesterone levels. After delivery the fall in progesterone levels restores gallbladder motility, with ejection of biliary sludge/stones through the bile duct, potentially leading to acute pancreatitis [21,22]. The increased incidence of acute pancreatitis only after delivery supports the role of gallbladder motility in the pathogenesis of acute pancreatitis.

In older and in breastfeeding mothers, longer amenorrhea and anovulatory cycles cause lower estrogen levels, likely resulting in lower bile cholesterol saturation, greater spontaneous stone dissolution and less acute pancreatitis cases [23,24]. In conclusion, in settings without ready access to tertiary surgical expertise for pregnancy-related abdominal surgery, our findings support UDCA as a pragmatic temporary bridge strategy after pregnancy-related gallstone pancreatitis to defer cholecystectomy until the post-partum period in selected patients with small stones and a patent cystic duct. Since recurrence after UDCA discontinuation cannot be excluded, laparoscopic cholecystectomy remains the preferred definitive treatment post-partum. UDCA may be considered only for women who strongly refuse surgery, with regular US surveillance (e.g., annual follow-up).

## Figures and Tables

**Table 1:** Clinical and ultrasonographic population data.

Patient	Age	No. of Stones/ Largest Diameter (mm)	AP in Pregnancy (Months)	AP Post-Partum (Months)	Months Since AP Stone Recurrence	Stone Dissolution (Months)	Follow-Up (Months)
1	27	1/4	5		0/0	4	72
2	24	1/7	7		0/0	6	84
3	19	2/6	6		0/0	9	72
4	20	1/7	7		0/0	9	88
5	23	3/8	7		84/84 *	6	72
6	39	1/9		2	0/0	9	72
7	21	1/4		4	0/0	9	72
8	18	2/9		2	0/0	12	72
9	26	1/8		7	0/0	5	84
10	29	1/4		3	0/0	3	98
11	33	1/9		5	0/0	12	92
12	25	2/7		11	1/1 **	0	1
13	23	1/3		2	0/0	5	72
14	36	1/9		9	0/0	12	76

Follow-up: 78.9 + 16.6 (mean ± SD) months (patients: *n* = 13) * relapse of stones and new AP 18 months since last follow-up visit. ** relapse of AP without GS dissolution.

## Data Availability

The original contributions presented in this study are included in the article. Further inquiries can be directed to the corresponding author.

## Abbreviations

Ursodeoxycholic Acid (UDCA), Transabdominal Ultrasonography (US), Computed Tomography (CT), Magnetic Resonance (MR).
